# Telomere-Mediated Chromosomal Instability Triggers TLR4 Induced Inflammation and Death in Mice

**DOI:** 10.1371/journal.pone.0011873

**Published:** 2010-07-29

**Authors:** Rabindra N. Bhattacharjee, Birendranath Banerjee, Shizuo Akira, M. Prakash Hande

**Affiliations:** 1 Akira Innate Immunity Project, Exploratory Research for Advanced Technology (ERATO), Osaka University, Osaka, Japan; 2 Department of Host Defense, Research Institute for Microbial Diseases, Osaka University, Osaka, Japan; 3 Department of Anatomy and Cell Biology, University of Western Ontario, London, Canada; 4 Department of Physiology, Yong Loo Lin School of Medicine, National University of Singapore, Singapore, Singapore; 5 World Premier International Immunology Frontier Research Center, Osaka University, Osaka, Japan; Roswell Park Cancer Institute, United States of America

## Abstract

**Background:**

Telomeres are essential to maintain chromosomal stability. Cells derived from mice lacking telomerase RNA component (mTERC^−/−^ mice) display elevated telomere-mediated chromosome instability. Age-dependent telomere shortening and associated chromosome instability reduce the capacity to respond to cellular stress occurring during inflammation and cancer. Inflammation is one of the important risk factors in cancer progression. Controlled innate immune responses mediated by Toll-like receptors (TLR) are required for host defense against infection. Our aim was to understand the role of chromosome/genome instability in the initiation and maintenance of inflammation.

**Methodology/Principal Findings:**

We examined the function of TLR4 in telomerase deficient *mTERC^−/−^* mice harbouring chromosome instability which did not develop any overt immunological disorder in pathogen-free condition or any form of cancers at this stage. Chromosome instability was measured in metaphase spreads prepared from wildtype *(mTERC^+/+^), mTERC^+/−^* and *mTERC^−/−^* mouse splenocytes. Peritoneal and/or bone marrow-derived macrophages were used to examine the responses of TLR4 by their ability to produce inflammatory mediators TNFα and IL6. Our results demonstrate that TLR4 is highly up-regulated in the immune cells derived from telomerase-null *(mTERC^−/−^)* mice and lipopolysaccharide, a natural ligand for TLR4 stabilises NF-κB binding to its promoter by down-regulating ATF-3 in *mTERC^−/−^* macrophages.

**Conclusions/Significance:**

Our findings implied that background chromosome instability in the cellular level stabilises the action of TLR4-induced NF-κB action and sensitises cells to produce excess pro-inflammatory mediators. Chromosome/genomic instability data raises optimism for controlling inflammation by non-toxic TLR antagonists among high-risk groups.

## Introduction

Telomeres are specialised DNA structures consisting of tandem arrays of short, repetitive G-rich sequences that are oriented in 5′-3′ direction towards the end of the chromosome. They preserve chromosome integrity and protect chromosomes from being recognised as damaged DNA. The end replication problem results in a gradual loss of telomeric sequence under normal conditions in cells lacking the enzyme telomerase [1], [2], [3], [4]. Activation of telomerase, a ribonucleoprotein, has been shown to be essential for the stable maintenance of telomere length *in vitro* and *in vivo*. Telomerase consists of an essential RNA component (in mouse called mTR or mTERC) which serves as a template for telomeric DNA synthesis and a catalytic protein (mTERT) component. This enzyme protects against chromosome end-to-end fusions not only by lengthening the telomere repeats but also by providing a cap at the end of the chromosome [4]. Telomere shortening, telomerase reverse transcriptase and telomerase hold important implications for ageing and oncogenesis.

Cells derived from mice lacking telomerase RNA component (mTERC^−/−^ mice) display elevated telomere-mediated chromosome instability [5], [6], [7]. Age-dependent telomere shortening and associated chromosome instability reduce the capacity to respond to cellular stress that occurs during inflammation and cancer [8], [9], [10]. Recently, it was shown that expression of interferon stimulated gene 15 (ISG15) increases in human cells with short telomeres [11]. It is speculated that upregulation of ISG15 with telomere shortening may contribute to chronic inflammatory states associated with human ageing [11]. In our recent studies [7], [12], mouse embryonic fibroblasts (MEFs) with dysfunctional telomeres derived from mTERC^−/−^ mice or from PARP-1^−/−^ mice displayed differential expression of genes involved in inflammatory response [5], [6], [13]. In addition, poly-(ADP-ribose) polymerase (PARP-1), a DNA break sensing molecule and a transcriptional co-factor for NF-κB-dependent gene expression, is also related to the immune response, which is implicated in almost all age-related or associated diseases [14]. Interestingly, microRNAs (mi-146a and mi-146b) were shown to negatively modulate the senescence-associated inflammatory mediators (IL-6 and IL-8) [15].

Infection and autoimmune disorders are commonly associated with inflammation and evidences support the notion that the presence of microbes can be a cofactor in tumour promotion and progression [16]. For example, there is a strong association between inflammatory bowel disease and colon cancer; infection with Helicobacter pylori and gastric cancer, chronic viral hepatitis and liver cancer [17]. Data from mouse models of human cancer have established that chronic inflammation creates a pro-tumour microenvironment that is an essential component of neoplastic processes [18], [19], [20]. The soluble inflammatory molecules secreted by tumour associated immune cells promote cell motility; induce angiogenesis and extravasation of tumour cells [21]. Interestingly, metastatic carcinoma cells secrete macrophage-activating factors leading to production of IL-6 and TNF-α through activation of TLR2 and 6 [20], [22]. However, till to date the precise mechanisms that link inflammation with tumour progression and metastasis remain to be elucidated.

Innate immune system is the body's first line of defense against infection. In vertebrates, the main function of innate immune receptors (Toll-like receptors or TLR) is to recognise the presence of foreign invaders in the form of pathogen associated molecular patterns (PAMPs,) on invading microorganisms and initiate downstream signals to produce inflammatory mediators as protective measures to defend hosts. The members of the TLR family are type 1 transmembrane proteins expressed primarily by certain cells of the innate immune system (e.g., macrophages, dendritic cells and neutrophils). TLRs and interleukin 1 receptors (IL-1Rs) share significant similarity in their cytoplasmic domain (called Toll-IL-1R or TIR domain. Typical ligands for the TLRs are microbial products such as bacterial unmethylated deoxycytidyl-deoxyguanosine (CpG) DNA, and lipopolysaccarides or viral RNA or nucleic acids. They mediate signals through several adaptor proteins such as MyD (myeloid differentiation factor) 88, MAL/TIRAP (MyD88-adaptor-like/TIR-associated protein), and TRIF (Toll-receptor-associated activator of interferon) to liberate pro- inflammatory transcription factor NF-κB [23], [24] which initiates robust transcription of most of the genes responsible for inflammation such as interleukin (IL)-6, cyclooxygenase (COX)-2, reactive oxygen species (ROS), TNF-α, etc as part of the tumour supportive micro-environment. Besides TLR's ability to recognise PAMPs, they also play an essential role in non-infectious sterile inflammation by recognising endogenous ligands from the damaged cells such as β-defensins, and oxidised lipids [25] or protein molecules modified by oxidation and nitration [23].

While innate immune responses via TLRs are absolutely required for host defence against infections, uncontrolled reaction from a TLR can also cause chronic inflammation eventually leading to cancer. Our aim was to understand the role of chromosome instability in the initiation and maintenance of inflammation. We examined the function of TLR4 in telomerase deficient *mTERC^−/−^* mice harbouring chromosome instability. At this stage, these mice did not exhibit any overt immunological disorder in pathogen-free condition or development of cancer. Genomic instability was measured in metaphase spreads obtained from wildtype *(mTERC^+/+^), mTERC^+/−^* and *mTERC^−/−^* mouse splenocytes. Peritoneal and/or bone marrow-derived macrophages were used to examine the responses of TLR4 by their ability to produce inflammatory mediators TNFα and IL6.

## Results and Discussion

**Figure 1:**
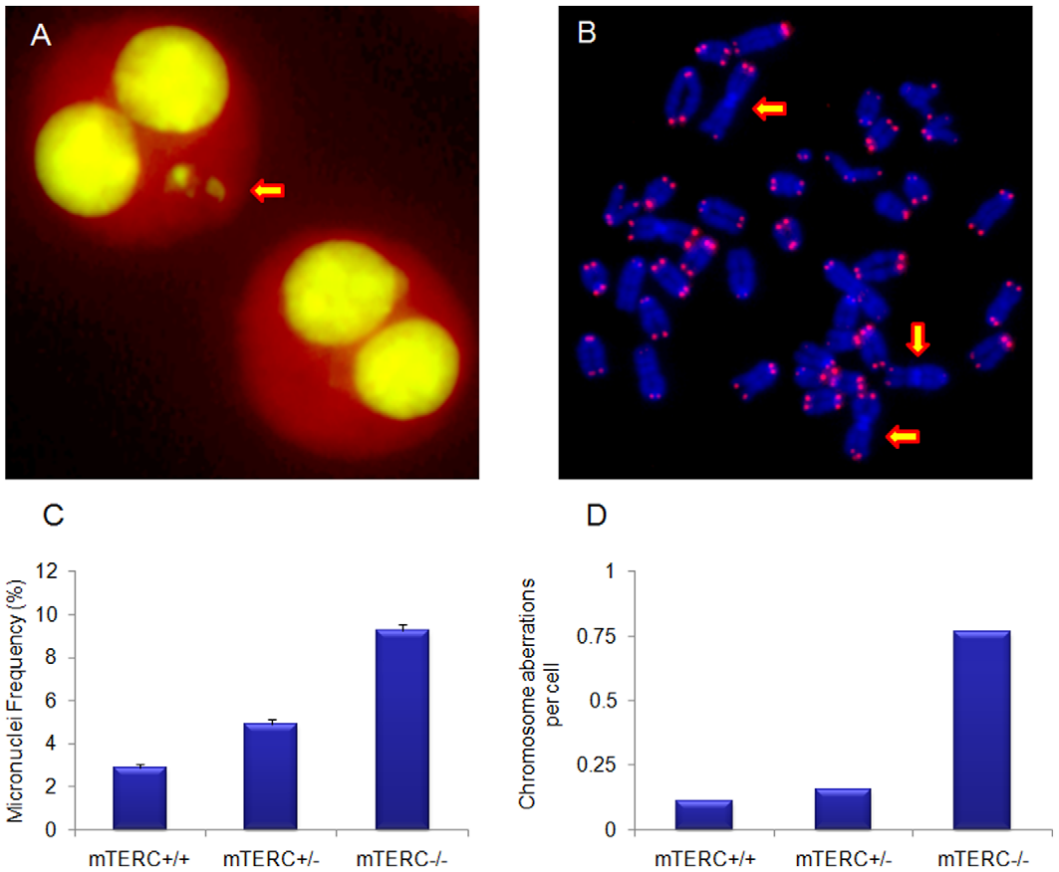
Genomic instability in *mTERC^−/−^* MEFs with dysfunctional telomeres. (**A**) Left arrow points a binucleated cell with two micronuclei, a marker for chromosomal instability. Right arrow points to a normal binucleated cell. (B) Telomere PNA-FISH on metaphase spreads from *mTERC^−/−^* MEFs. Chromosomal DNA was stained with DAPI (blue) and telomeres were hybridised with Cy3-labeled telomere probe (pink). Left arrows point Robertsonian fusion like configuration (RLC) and down arrow indicates a dicentric chromosome (DC) representing chromosomal aberrations. **C**). Histogram showing number of micronuclei measured by the CBMN assay D) Histogram showing total chromosome aberrations detected per cell in different cell types.

**Figure 2:**
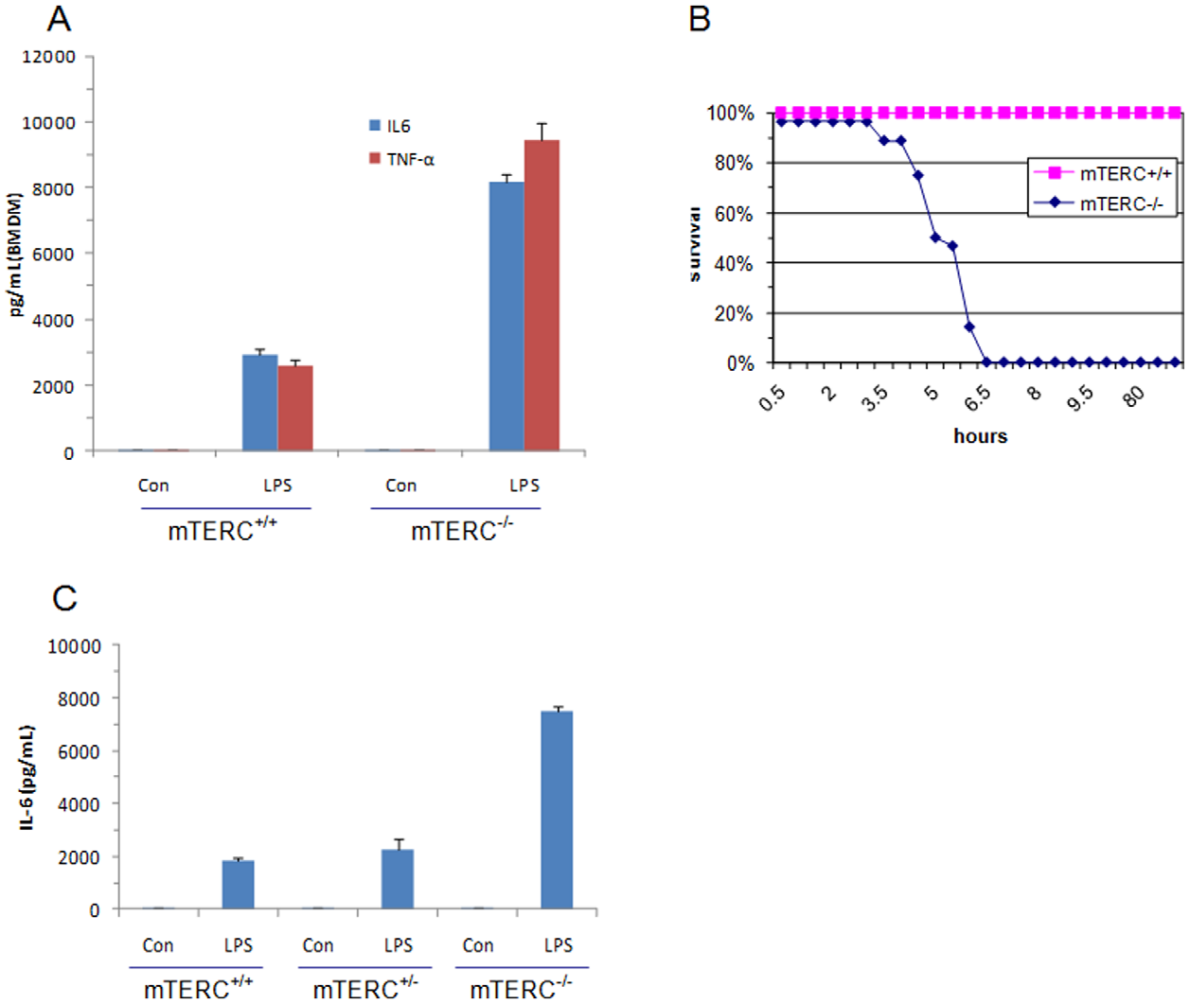
TLR4 induced inflammation in *mTERC^+/+^* and *mTERC^−/−^* mice. (A) Upon LPS stimulation of *mTERC* ***^+/+^*** and *mTREC^−/−^* BMDMs, IL6 and TNFα levels were determined by ELISA (right) (**B**) Age-matched *mTERC* ***^+/+^*** and *mTERC^−/−^* littermates (n = 8) were administered intraperitoneally with LPS (1 µg/g body weight) and monitored for survival. (C) Sera were collected after 2 h of LPS injection from different group of mice (n = 3) and cytokine levels determined by ELISA (right).

Absence of active telomerase in mice resulted in progressive telomere shortening both *in vivo* and *in vitro* [5], [6], [26]. Short or dysfunctional telomeres produced massive chromosome/genomic instability in these cells [5], [6]. In our earlier study, we have observed that the cells with dysfunctional telomeres are specifically sensitive to oxidative damage [7]. A similar response to oxidative stress was detected in mouse cells lacking a DNA break sensing molecule, PARP-1 [13] and PARP-1^−/−^ MEFs display abnormal telomeres and chromosome instability. Microarray analysis on mTERC^−/−^ and PARP-1^−/−^ MEFs revealed differential expression of genes involved in immune response (unpublished). In the present study, we sought to determine the link, if any, between telomere mediated chromosome/genomic instability and initiation and maintenance of inflammation. Genomic instability, quantified following telomere staining, includes micronuclei induction (Fig. 1A), dicentric chromosomes and Robertsonian-fusion-like configurations (Fig. 1B). Percentage of micronuclei (Fig. 1C) and chromosomal aberrations (Fig. 1D) in the splenocytes were three times higher in *mTERC^−/−^* mice compared to *mTERC^+/+^* mice. Although *mTERC^+/+^* and *mTERC^+/−^* macrophages produced normal level of TNFα-IL6 in response to lipopolysaccharide (LPS), a 6–12 fold induction of IL6 and TNFα have been observed in *mTERC^−/−^* macrophages (both in peritoneal exudate cells and bone marrow-derived macrophages) (Fig. 2A). Progressive telomere shortening was seen in *mTERC^−/−^* cells in culture [6], [7] and in *mTERC^−/−^* mice [26]. To find out any contribution of telomere shortening in TLR4 action, we incubated PD80 (∼37 kb; PD - Population Doubling) and PD200 (∼16 kb) MEFs with LPS and measured TNF-α and IL6. Consistent with the *mTERC^−/−^* bone marrow-derived macrophages, PD 200 MEFs produced 4–7 fold increase of IL6 and TNF-α as compared to PD 80 (data not shown). Using microarray analysis, Lou *et al.* [11] demonstrated that upregulation of ISG15, an interferon stimulated gene which has cytokine-like immunomodulatory properties [27], correlates with telomere shortening in human cells. This increase in the level of ISG15 was not genotoxic stress dependent but correlated with telomere length [11].

To clarify the *in vivo* function of TLR4 in chromosome instability, we produced systemic inflammation in age-matched WT and *mTERC^−/−^* mice by intra-peritoneal LPS injection. Notably, *mTERC^−/−^* mice exhibited significant reduction of survival (succumbed between 3.5 h to 6.5 h after LPS administration) than WT littermate (none expired within 100 h period) (Fig. 2B). Death of *mTERC^−/−^* mice was relevant to severe endotoxin shock and subsequent systemic inflammatory reaction as their serum circulating levels of IL6 and TNF-α reached greater than 4-fold within 2 h of LPS injection and 1.5 h before the first death (Fig. 2C).

**Figure 3:**
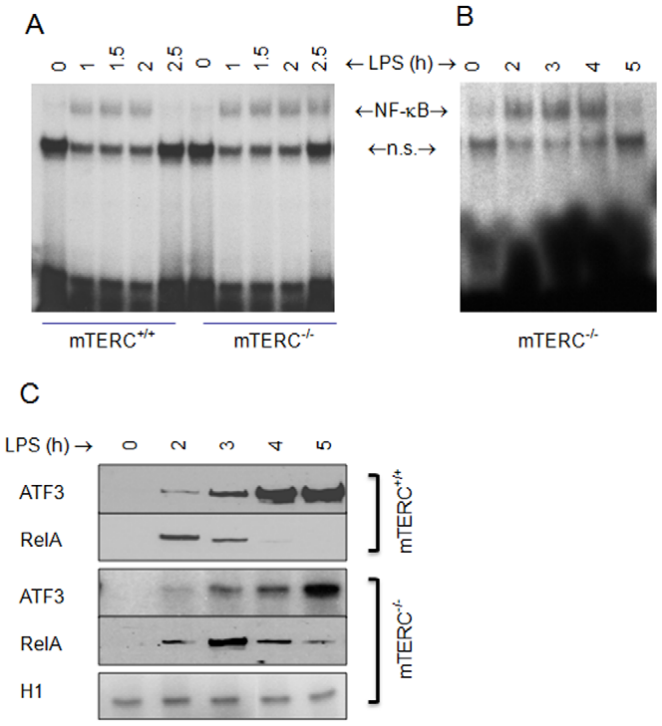
TLR4 induced inflammation in *mTERC^+/+^* and *mTERC^−/−^* mice. (A) and (B) BMDMs from *mTERC* ***^+/+^*** and *mTERC^−/−^* mice were stimulated with 10 ng/mL LPS for the indicated times. Nuclear extracts were subjected to EMSA using NF-κB probe. (C) Nuclear extracts above were also subjected to western blot for ATF3 and Rel (p65 subunit of NF-κB). Histone H1 was used as a loading control (right).

TLR4-induced transcription of IL-6 is closely controlled by the nuclear factor (NF)-κB [28]. To determine the specific role of NF-κB in the differential immune regulation in WT and *mTERC^−/−^* bone marrow-derived macrophages, we performed NF-κB DNA binding assays with nuclear extract from these cells at several time points. NF-κB binds with equal intensity to the TNF-α promoter probe within 1 h of LPS stimulation of the WT and *mTERC^−/−^* bone marrow-derived macrophages. In *mTERC* ***^+/+^*** cells, this binding disappears within 2.5 h. In contrast, binding of *mTERC^−/−^* NF-κB remains constant for at least 4 h and only started declining after 5.3 h of LPS stimulation (Fig. 3A and B). We have identified the mechanism of this longer half-life of NF-κB in chromosome instability as the function of activating transcription factor (ATF)-3. We measured the relative nuclear concentration of Rel A (p65 subunit of NF-κB) and ATF-3 in the nuclear extracts, which we used for NF-κB DNA binding assays. While LPS-induced augmentation of Rel A persisted up to 4 h in WT cells, that in *mTERC^−/−^* cells remained steady until 5.3 h (Fig. 3C). An increasing amount of ATF-3 was consistently correlated with the decreasing amount of Rel A at all time points, indicative of negative regulatory function of ATF-3 in LPS-induced inflammation and chromosomal instability.

Many cancer cells do not metastasise because successful metastasis requires cancer cell specific intrinsic factors as well as extrinsic factors derived from tumour microenvironment. In these cases, we believe that mutational events during the process of carcinogenesis select out cells with activated TLRs that can orchestrate the production of inflammatory mediators (IL-1, IL-6, TNF-α etc.) and recruits immune cells overloaded with TLRs. In combination, they orchestrate a signalling cascade leading to overproduction of many molecules such as angiogenic factors that facilitate cancer progression and metastasis. While exploring the connection between chromosomal instability and immune responses, we have now established the obligatory role of toll-like receptors. Our findings implicate that background chromosome instability in the cellular level stabilises the action of TLR4-induced NF-κB action and sensitises cells to produce excess pro-inflammatory mediators. Chromosome instability status may be used as a prognostic indicator because it has potential to orchestrate an inflammatory microenvironment for many progressive diseases including cancers. The finding that TLR4 action correlates to the telomere length and associated chromosome instability in mouse cells suggests that telomeres are involved in broader immune functions beyond the protection of chromosomes. We explained one of the root causes of the inflammation by linking host's telomere-dependent chromosomal status and deregulation of immunity. It raises optimism for controlling inflammation by non-toxic TLR antagonists among high-risk groups.

## Materials and Methods

### Ethics Statement

The animal breeding protocol as well as the experiments conducted in this study are approved by the Institutional Animal Care and Use Committee (IACUC) of the National University of Singapore (IACUC Protocol 755/05 and 828/05).

### Reagents and cells

LPS from *E.coli* serotype 055:B5 (phenol extracted and then chromatograpically purified by gel filtration) was purchased from Sigma. LPS was solubilised in distilled water by sonication. Bone marrow was collected from femurs in complete RPMI with 10% heat-inactivated FBS, 2 mM glutamine, 100 U/ml penicillin, 100 ug/ml streptomycin and 50 ng/ml rmM-CSF. Resident peritoneal exudate cells from wild type and *mTERC^−/−^* mice were isolated from the peritoneal cavity of mice 3 d after injection with 2 ml of 4% thioglycollate medium (Sigma) and were cultured in RPMI1640 supplemented with 10% foetal bovine serum. Both peritoneal exudate cells and bone marrow-derived macrophages were stimulated with LPS (10 ng/ml) for the indicated times and production of IL6 and TNFα were measured by enzyme-linked immunosorbent assay (ELISA).

### Florescence *in Situ* Hybridisation (FISH) with Peptide Nucleic Acid-telomere Probe

Plates containing primary MEFs or established cell lines were treated with colcemid (0.1 µg/ml) for 4 - 5 h and subsequently trypsinised and spun for 8 min at 120 g. After hypotonic swelling in sodium citrate (0.03 M) for 20 min at 37°C, cells were fixed in methanol/acetic acid (3∶1). After 2–3 additional changes of fixative, cell suspensions were dropped on wet, clean slides and dried overnight. FISH with Cy-3 labelled (CCCTAA)_3_ peptide nucleic acid, and subsequent quantitative analysis of digital images were performed [6].

### Cytokinesis-blocked micronucleus assay

Chromosome instability was also measured by cytokinesis-blocked micronucleus assay [29], [30]. Cells were incubated with cytochalasin B (Sigma, 5 µg/ml) for 22 h. The cells were then trypsinised and subsequently fixed using a combination of both Carnoy's fixative (acetic acid/methanol 1∶3) and three to four drops of formaldehyde (to fix the cytoplasm). Fixed cells were dropped onto clean slides and stained with 3 µg/ml of acridine orange, which differentially stains cytoplasm and nucleus [31], [32]. One thousand binucleated cells were scored for each sample.

### Measurement of proinflammatory cytokine concentrations

Primary peritoneal macrophages, bone marrow-derived macrophages, and MEFs were cultured with 50 ng/ml of *Escherichia coli* LPS for 4–8 h. To produce systemic inflammation in mouse, LPS (1 µg/g body weight) was given by intraperitoneal injection. Blood was obtained by retro-orbital puncture after anaesthesia with methoxyflurane, and the mice were asphyxiated with carbon dioxide. Survival of the mouse was monitored for one week post injection of LPS. Concentration of TNFα and IL6 in culture supernatants and serum were measured by ELISA according to manufacturer's instructions (R & D Research systems).

### Electrophoretic Mobility shift assay

The nuclear extracts of bone marrow derived macrophages (5×10^6^) were purified after LPS stimulation. The extract was incubated with a specific probe for NF-κB DNA binding site, electrophoresed, and visualised by autoradiography.

### Western Blot analysis

Purified nuclear extracts used in electrophoretic mobility shift assay were resolved by SDS-PAGE (40 µg total protein) and transferred onto a polyvinylidene fluoride membrane. The membrane was blotted with the specific antibodies to indicated proteins and visualised with an enhanced chemiluminescence system (NEN life science).
